# Supplementary material for: A fine-grained evaluation framework for urban land cover change based on feature monitoring with remotely sensed imagery
Source: PLoS One. 2026 Feb 23;21(2):e0342350. doi: 10.1371/journal.pone.0342350 (PMC12928421; doi:10.1371/journal.pone.0342350)
Supplement: S1 Table — (DOCX) [file pone.0342350.s001.docx]

| **Algorithm 1: Urban Adaptive Shared-feature Attention (UASF) Module.** |
| --- |
| Input: Feature map X ∈ R^B×C×H×W^  Parameters: DWConv, PWConv, Sobel(·), FC1, FC2, Proj(·), σ(·), δ(·)  Output: Fused feature representation Y∈ R^B×C×H×W^  1: % Semantic Embedding Branch (Feature-domain)  2: E ← PWConv(DWConv(X))  3:  4: % Structural Modulation Branch (Gradient-domain)  5: Gx, Gy ← Sobel(X)  6: S ← sqrt(Gx ⊙ Gx + Gy ⊙ Gy + ε)  7: S ← Normalize(S)  8: 9: % Semantic-Structural Consistency Kernel  10: Z ← E ⊙ (1 + S) (⊙: element-wise multiplication)  11: Z ← Proj(Z)  12:  13: % Global Adaptive Gating  14: v ← GAP(X)  15: a ← δ(FC1(v))  16: g ← σ(FC2(a))  17: g ← Reshape(g) to R^B×C×1×1^  18:  19: % Final Fusion Output  20: Y ← g ⊙ Z + (1 − g) ⊙ X  21: return Y |
