# Supplementary material for: A fine-grained evaluation framework for urban land cover change based on feature monitoring with remotely sensed imagery
Source: PLoS One. 2026 Feb 23;21(2):e0342350. doi: 10.1371/journal.pone.0342350 (PMC12928421; doi:10.1371/journal.pone.0342350)
Supplement: Attachment — Submitted filename: PONE-D-25-61989_Report.docx [file pone.0342350.s002.docx]

**A Fine-grained Evaluation Framework for Urban Land Cover Change Based on UASFNet Data-driven Approach**

The article presents a study on urban land cover change using a novel **data-driven framework** based on the **UASFNet model**, which leverages deep learning techniques for high-precision urban land cover classification. As urbanization and climate change intensify, understanding land cover dynamics becomes increasingly important for urban planning and ecological conservation.

1. All figures should be improved in term of map elements delivery and clarity. For each figure, include clear captions contextualizing the data presented, methodologies used for analysis, and key takeaways. e.g. Fig. 3 it is difficult to identify difference between others and road class. I suggest mark road as red/grey and others class as sand color.

Figure 1 is difficult to understand. Only there are two steps highlighted, flow is not symmetrical.

2. How does the proposed UASFNet model compare to traditional methods in terms of accuracy and computational efficiency?
3. In **2.1 Fine-Scale Assessment Framework (Lines 106-113)**, what specific preprocessing techniques were applied to the bi-temporal remote sensing images? Could this impact the overall accuracy of the analysis?
4. For **2.2 UASFNet Model (Lines 117-164)**, how does the shared-feature attention mechanism operate in varying urban contexts? Are there limitations to its adaptability?
5. **(Lines 165-171)** Why were the specific evaluation metrics chosen, and do you believe they comprehensively reflect the model's performance?
6. How does the study handle edge cases where certain categories of land cover may have limited training data?
7. **(Lines 285-315) w**hat are the specific computational complexities of UASFNet compared to the other models presented in **Table 5**? How are computational costs justified relative to the performance gains?
8. In **Table 2**, what specific criteria were used to classify the models into "mainstream," and could this bias the results?
9. **(Lines 324-372)** In **Figure 5**, how were the multi-temporal classification results verified? What controls or validation processes were in place during the mapping?
10. For **Figure 6** and the corresponding discussion, what were the specific methodologies used to calculate the changes in impervious surfaces and green space? Could there have been biases in the data collection that affect these classifications?
11. Regarding **Figure 7**, can you elaborate on the significance of the spatial distribution patterns observed? How might these patterns inform future urban planning initiatives?
12. **(Section 5, Lines 451-472)** How can the findings of this study potentially influence policy on urban land use? What limitations in the data could potentially hinder this influence?
13. What future applications are envisioned for the UASFNet framework, and how will evolving technology be incorporated into novel methodologies?
