# Supplementary material for: A fine-grained evaluation framework for urban land cover change based on feature monitoring with remotely sensed imagery
Source: PLoS One. 2026 Feb 23;21(2):e0342350. doi: 10.1371/journal.pone.0342350 (PMC12928421; doi:10.1371/journal.pone.0342350)
Supplement: Attachment — Submitted filename: Response to Reviewers.docx [file pone.0342350.s004.docx]

**Thanks for all comments and** **suggestions of the reviewers. They are all significant for our research work and paper writing.** **We have a** **detailed revision of this paper** **and changes are marked in yellow. The** **presentation in the revised paper has been improved. Now we will response to the comments as follows.**

**Response to Academic Editor:**

(1) *Q: Please ensure that your manuscript meets PLOS ONE's style requirements, including those for file naming.*

A: We have carefully revised the manuscript to ensure full compliance with PLOS ONE’s style and formatting requirements, including file naming conventions. The revised submission follows the official PLOS ONE templates for the main text and title/authors/affiliations, and all required files have been correctly prepared and labeled.

(2) *Q: Please provide additional details regarding participant consent.*

A: This study involved expert consultation for constructing the Analytic Hierarchy Process (AHP) judgment matrix. Informed verbal consent was obtained from all participating experts prior to data collection. Participation was voluntary, and no personal, sensitive, or identifiable information was collected. According to institutional guidelines, formal ethical approval wasnot required for this type of low-risk expert consultation.

(3) *Q: Please note that PLOS One has specific guidelines on code sharing for submissions in which author-generated code underpins the findings in the manuscript.*

A: We have made the author-generated code underpinning the findings of this study publicly available in accordance with PLOS ONE’s code sharing policy. The implementation of the proposed UASFNet framework, including the UASF module described in Section 2.3 of the manuscript, has been uploaded to a public GitHub repository at https://github.com/lazy0cat0orange/UASFNet. The shared code provides a research-oriented implementation sufficient to reproduce the core methodology and experimental results reported in the manuscript.

(4) *Q: We note that the grant information you provided in the ‘Funding Information’ and ‘Financial Disclosure’ sections do not match.*

A: We acknowledge the inconsistency in the funding information in the initial submission. This has now been corrected. In accordance with PLOS ONE’s manuscript organization guidelines, the funding information has been removed from the manuscript text and has been accurately and consistently updated in the online submission system (Funding Information and Financial Disclosure), which should be considered the authoritative source.

(5) *Q: Please include both an updated Funding Statement and Competing Interests Statement in your cover letter.*

A:We have updated the Funding Statement and Competing Interests Statement to fully disclose the commercial affiliation of some authors with Tianjin Huashui Engineering Consulting Co., Ltd. The revised statements clarify that the funder provided support in the form of salaries only and had no role in the study design, data collection and analysis, decision to publish, or preparation of the manuscript. We also confirm that this affiliation does not alter our adherence to all PLOS ONE policies on sharing data and materials.

(6) *Q: In this instance it seems there may be acceptable restrictions in place that prevent the public sharing of your minimal data.*

A: Data access requests may be directed to the Data Management Office of Tianjin Huashui Engineering Consulting Co., Ltd. (contact email: tjhuashui_data@163.com), which is independent of the authors and responsible for coordinating data access and ensuring long-term data stewardship on behalf of the institution. Access will be considered upon reasonable request and subject to institutional approval.

(7) *Q: We note that Figures 2, 3, 4, 5, 6, 7, 8, and 9 in your submission contain map/satellite images which may be copyrighted.*

A: We have carefully reviewed all figures containing maps or satellite imagery. All potentially copyrighted materials have been removed or replaced. The revised figures are based exclusively on publicly available datasets or author-generated visualizations, and fully comply with the CC BY 4.0 licensing requirements of PLOS ONE.

“Fig 1. Overall framework of the proposed fine-scale urban land cover change assessment method. All elements were created by the authors.

Fig 2. Location map of the study area. (A) the Langfang dataset (China). (B) the Potsdam dataset (Germany). (C) the Guiyang dataset (China). The global location map was generated using the Natural Earth dataset, which is in the public domain. Representative remote sensing imagery was obtained from the Copernicus Data Space Ecosystem (Copernicus Sentinel data), the ISPRS Potsdam dataset and processed by the authors. All map elements and annotations were created by the authors.

Fig 3. Example image patches and corresponding ground truth labels from the Potsdam dataset. The figure was generated by the authors for visualization purposes.

Fig 4. Visual comparison of urban land cover classification results produced by different models on the the Potsdam dataset. All classification results were generated by the authors.

Fig 5. Multi-temporal urban land cover classification results for the Langfang Economic Development Zone. The background remote sensing imagery was obtained from the Copernicus Data Space Ecosystem (Copernicus Sentinel data) and processed by the authors. The imagery represents original satellite observations rather than an online basemap. All classification maps, boundaries, annotations, and map elements were generated by the authors.

Fig 6. Comparative charts of multiple indicators across the 34 subregions of the Langfang Economic Development Zone (2017–2023). All map elements were generated by the authors.

Fig 7. Spatial distribution of individual urban land cover disturbance indicators. All thematic maps were produced by the authors.

Fig 8. Spatial distribution of comprehensive urban land cover change intensity. All visual elements were generated by the authors.

Fig 9. Large range of recognition effect comparison diagram in Langfang. Background imagery was obtained from the Copernicus Data Space Ecosystem(Copernicus Sentinel data) and processed by the authors for visualization and comparison. The imagery corresponds to original satellite data and is not derived from any third-party online basemap or proprietary map service. All classification results, overlays, and map elements were generated by the authors.”

**Response to reviewer #1:**

(1) *Q: Expand the study to include cities with different topographies, such as mountainous cities like Guiyang.*

A: We sincerely appreciate this valuable suggestion. We agree that including cities with diverse topographies is crucial for validating the robustness and generalization ability of our method. Following your advice, we have expanded our study to include the Guiyang Dataset, which represents a typical mountainous urban environment. This addition ensures our study covers a broader range of geographical scenarios.

The revisions have been made in Section 2.2, 3.1 (Lines 147-150, 348-353) of the revised manuscript. The added description is as follows:.

“(c) Guiyang Dataset: This dataset covers the urban core area of Guiyang City, China, representing a typical mountainous urban environment. It was constructed following the same data preparation, annotation protocol, and data partition strategy as the Langfang dataset, ensuring consistency and comparability across datasets.

On the Guiyang dataset, UASFNet achieved the best overall performance, with an mIoU of 88.90%, an F1-score of 93.65%, and a Kappa value as high as 0.9493. Among the four land-cover categories, the IoU values for buildings, roads, and greenbelts reached 96.58%, 96.44%, and 94.62%, respectively.

Table 2 Quantitative performance comparison of different semantic segmentation models on three urban remote sensing datasets.

| Dataset | Method | IoU(%) per Category | | | |  | Metrics | | | |
| --- | --- | --- | --- | --- | --- | --- | --- | --- | --- | --- |
|  |  | Building | Greenbelt | Road | Others |  | Accuracy | MF1 | MIoU | Kappa |
| Guiyang Dataset | ResNet | 96.35 | 96.2 | 94.46 | 66.32 |  | 96.27 | 93.28 | 88.33 | 0.9468 |
|  | UNetformer | 96.32 | 96.22 | 94.56 | 67.86 |  | 96.32 | 93.56 | 88.74 | 0.9476 |
|  | CMTFNet | 95.69 | 96.07 | 92.71 | 64.94 |  | 95.73 | 92.69 | 87.35 | 0.9392 |
|  | CM-Unet | 95.65 | 96.09 | 93.76 | 65.71 |  | 96.00 | 92.97 | 87.80 | 0.9429 |
|  | MFNet | 95.07 | 96.24 | 93.79 | 66.05 |  | 95.91 | 92.98 | 87.79 | 0.9419 |
|  | UASFNet (Ours) | 96.58 | 96.44 | 94.62 | 67.95 |  | 96.45 | 93.65 | 88.90 | 0.9493 |

”

(2) *Q: On line 256, the ratio of training to validation data is not specified. Please clarify this key experimental detail.*

A: We thank the reviewer for pointing out this oversight. We apologize for not specifying the ratio of training to validation data in the original manuscript. To address this, we have explicitly clarified the data partition strategy and the specific quantity of data for both the Langfang and Potsdam datasets in the revised version.

Specifically, we specified a ratio of 70% / 10% / 20% (training/validation/test) for the Langfang dataset and provided the exact number of image patches for the Potsdam dataset. The revisions can be found in Section 2.2.2 (Lines 136-146) of the revised manuscript:

“(a) Langfang Dataset: This dataset was constructed using RGB three-band remote sensing images of the Langfang Economic Development Zone with a spatial resolution of 0.6 m. Four land-use classes were annotated using GIS software, and the dataset was partitioned into training, validation, and test sets using a spatial block segmentation method at a ratio of 70% / 10% / 20%, respectively.

(b) Potsdam Dataset: This dataset is derived from ultra–high-resolution TOP imagery with a ground sampling distance (GSD) of 5 cm. The Potsdam region is known for its complex building layouts and dense urban structures. The dataset covers an area of 3.42 km² and includes pixel-level annotations for six semantic categories. It has become a standard benchmark for semantic segmentation research. In this study, an improved four-class semantic labeling scheme—including buildings, greenbelts, roads, and others—was adopted to better suit urban analysis tasks, resulting in 2299 training, 605 validation, and 1694 test image patches of size 1024 × 1024.”

(3) *Q: For large-area identification, even with high-resolution imagery, errors are inevitable. However, the paper does not address the error characteristics of the reference data, which is critical for evaluating the reliability of the proposed method.*

A: This is a highly insightful and critical comment. We fully agree with the reviewer that addressing the error characteristics of the reference data is essential for properly evaluating the reliability of the proposed method. To address this concern, we have added a discussion acknowledging that, as with most large-area urban mapping studies, reference labels are subject to unavoidable uncertainties, particularly in transitional zones, complex building boundaries, and shadow-affected areas. Furthermore, we clarified that since all comparative models were trained and evaluated using the same reference data and partitioning strategy, these uncertainties affect all methods consistently and do not compromise the validity of the relative performance comparison.

The revisions can be found in Section 2.4 (Lines 230-234) of the revised manuscript:

“It should be noted that, as in most large-area urban land cover mapping studies, reference labels are subject to unavoidable uncertainties, particularly in transitional zones, complex building boundaries, narrow road segments, and shadow-affected areas. Since all models were trained and evaluated using the same reference data and partitioning strategy, such uncertainties affect all methods consistently and do not compromise the validity of the relative performance comparison.”

(4) *Q: The discussion section requires revision. Since five algorithms are mentioned, it would greatly enhance the persuasiveness of your work to compare your algorithm with these existing methods using the same study areas reported in their respective papers.*

A: We greatly appreciate the reviewer’s suggestion regarding the comparative analysis. We understand that evaluating algorithms on their original study areas can provide context. However, direct comparisons across different original datasets can introduce significant bias due to variations in image sources, resolutions, annotation standards, and partition strategies.

To ensure the most rigorous and fair comparison, our strategy was to evaluate all five algorithms under identical experimental settings (using the same datasets and data partition protocols). This approach eliminates the interference of data heterogeneity and allows for a more accurate assessment of the intrinsic performance differences between the models. We have clarified this rationale in the revised manuscript to emphasize the validity of our controlled comparison. The revisions can be found in Section 4.1 (Lines 488-489) of the revised manuscript:.

“All models were trained and evaluated on the same datasets and data partitions to ensure a controlled and fair comparison across different urban scenarios.”

**Response to reviewer #2:**

(1) *Q: All figures should be improved in term of map elements delivery and clarity. For each figure, include clear captions contextualizing the data presented, methodologies used for analysis, and key takeaways. e.g. Fig. 3 it is difficult to identify difference between others and road class. I suggest mark road as red/grey and others class as sand color. Figure 1 is difficult to understand. Only there are two steps highlighted, flow is not symmetrical.*

A: Thank the reviewers for their detailed suggestions regarding the quality of the charts, we have redrawn the relevant diagrams in the text, with a particular focus on enhancing the expression of map elements and improving the overall clarity. The specific modifications are as follows:

Regarding Figure 1: We have redesigned the structure of the flowchart, optimized the highlighting of steps, and ensured that the overall process is more symmetrical and the logic is more coherent, so that readers can understand our technical route more easily.

Regarding Figure 3: We have adopted your suggestion and adjusted the color scheme, enhancing the contrast between the categories (especially between the roads and other categories) to ensure a clear visual distinction.

The updated figures and captions can be found in Section 2 of the revised manuscript. The revised captions are as follows:.

“

Fig. 1 Overall framework of the proposed fine-scale urban land cover change assessment method.

Fig. 3 Example image patches and corresponding ground truth labels from the Potsdam dataset.”

(2) *Q: How does the proposed UASFNet model compare to traditional methods in terms of accuracy and computational efficiency?*

A: We thank the reviewer for this important question. we have provided a detailed comparative analysis in the revised manuscript (see Table 5). The results indicate that, from the perspective of model efficiency and performance balance, UASFNet demonstrates clear advantages over existing deep learning–based segmentation approaches. Specifically, our model achieves superior segmentation accuracy without relying on excessively large parameter sizes or computational costs. This balanced performance is primarily attributed to the Urban Adaptive Shared-feature Attention (UASF) module, which promotes effective semantic information sharing across multiple land cover categories while suppressing redundant feature propagation. As a result, UASFNet is able to extract more compact and discriminative representations, improving recognition performance under comparable computational conditions.

The revisions and detailed discussions can be found in Section 4.1 (Lines 481-488) and Table 5 of the revised manuscript..

“From the perspective of model efficiency and performance balance, UASFNet demonstrates clear advantages over existing deep learning–based segmentation approaches. As summarized in Table 5, the proposed model achieves superior segmentation accuracy without relying on excessively large parameter sizes or computational costs. This balanced performance can be attributed to the Urban Adaptive Shared-feature Attention (UASF) module, which promotes effective semantic information sharing across multiple land cover categories while suppressing redundant feature propagation. As a result, UASFNet is able to extract more compact and discriminative representations, improving recognition performance under comparable computational conditions.

Table 5 Model Efficiency and Performance Comparative Analysis

| Method | FLOPs (G) | Param.(M) | mIoU (%) |
| --- | --- | --- | --- |
| ResNet | 23.5 | 47.43 | 84.41 |
| UNetformer | 30.17 | 61.59 | 87.34 |
| CMTFNet | 76.41 | 96.14 | 90.30 |
| CM-Unet | 33.26 | 64.02 | 89.27 |
| MFNet | 54.39 | 67.72 | 87.45 |
| UASFNet (Ours) | 30.925 | 79.88 | 91.52 |

”

(3) *Q: In 2.1 Fine-Scale Assessment Framework (Lines 106-113), what specific preprocessing techniques were applied to the bi-temporal remote sensing images? Could this impact the overall accuracy of the analysis?*

A: We thank the reviewer for the inquiry regarding preprocessing details. We agree that specifying these steps is essential for reproducibility and reliability.

In the revised manuscript, we have explicitly detailed the preprocessing techniques applied. Specifically, to ensure consistency across multi-source and multi-temporal imagery, we performed geometric co-registration, spatial resampling to a unified resolution, and RGB value normalization. Regarding the potential impact on accuracy, we clarified that we deliberately avoided aggressive radiometric correction to preserve original spatial patterns. Furthermore, by applying this identical preprocessing pipeline to all datasets and baseline models, we minimize bias and ensure a fair and unbiased comparison.

The revisions can be found in Section 2.2.2 (Lines 151-157) of the revised manuscript:

“For all datasets, standard preprocessing procedures were applied prior to model training and inference to ensure consistency across multi-source and multi-temporal imagery. These procedures include geometric co-registration between different acquisition periods, spatial resampling to a unified spatial resolution, and normalization of RGB values. No aggressive radiometric correction or handcrafted feature extraction was introduced, in order to preserve original spatial patterns. The same preprocessing pipeline was applied to all datasets and baseline models to ensure a fair and unbiased comparison.”

(4) *Q: For 2.2 UASFNet Model (Lines 117-164), how does the shared-feature attention mechanism operate in varying urban contexts? Are there limitations to its adaptability?*

A: We thank the reviewer for this insightful question regarding the core mechanism of our model. Discussing the adaptability and boundary conditions of the attention mechanism is crucial for understanding the model's reliability.

In the revised manuscript, we have elaborated on the operational principle of the UASF mechanism: it operates in a data-driven manner to learn attention weights, thereby automatically focusing on semantic and structural patterns that remain consistent across varying urban contexts (such as building boundaries, road connectivity, and greenbelt textures).

In the discussion section, relevant explanations were also provided. When encountering significant "domain shifts" (such as huge differences in architectural styles, severe shadow effects, or highly heterogeneous surface materials), the adaptability of the model may be limited.

The revisions and discussion can be found in Section 4.1 (Lines 493-502) of the revised manuscript:

“The semantic–structural consistency kernel encourages the alignment of semantic representations with geometric cues, which is beneficial for preserving object boundaries and reducing confusion between visually similar land cover classes. The gradient-domain formulation highlights local structural variations, such as edges and shape transitions, that are particularly relevant for fine-scale urban patterns. The global gating operator adaptively modulates the relative contributions of semantic and structural information across spatial locations, thereby promoting spatial coherence while limiting redundant feature propagation.

The performance advantages of UASFNet can be attributed to the Urban Adaptive Shared-feature Attention (UASF) mechanism. This mechanism is designed to emphasize semantic and structural patterns that are consistently informative across different urban contexts, such as building boundaries, road connectivity, and greenbelt textures. Because the attention weights are learned in a data-driven manner rather than being manually specified, the mechanism can adapt to variations in urban morphology, density, and spatial configuration across different study areas. Nevertheless, its adaptability may be constrained under conditions involving pronounced domain shifts, such as cities with markedly different architectural styles, severe shadow effects, or highly heterogeneous surface materials. In such cases, incorporating additional domain-specific supervision or multi-source auxiliary information may further enhance robustness, which is considered a direction for future work.”

(5) *Q: (Lines 165-171) Why were the specific evaluation metrics chosen, and do you believe they comprehensively reflect the model's performance?*

A: We thank the reviewer for the question regarding the selection of evaluation metrics. We fully agree that a well-justified combination of metrics is essential for a comprehensive and objective assessment of model performance.

To address this, we have elaborated on the scientific rationale behind our choices in the revised manuscript. Specifically, we employed mIoU as the primary metric for quantifying overall pixel-level accuracy. This is complemented by Precision, Recall, and F1-score to characterize class-wise discrimination, which is particularly critical for handling imbalanced or structurally complex categories common in urban scenes. Furthermore, we included the Kappa coefficient to evaluate the agreement between predicted and reference maps beyond random chance.

We believe that this suite of metrics comprehensively reflects the model's performance by capturing pixel-wise accuracy, class-wise consistency, and statistical robustness. This ensures that the resulting land-cover maps serve as reliable inputs for the subsequent urban disturbance analysis. The revisions can be found in Section 2.4 (Lines 217-224) of the revised manuscript:

“The mean Intersection over Union (mIoU) was used as the primary metric to quantify overall pixel-level classification accuracy across land-cover classes. Precision, recall, and F1-score were employed to further characterize class-wise discrimination performance, particularly for imbalanced or structurally complex categories. In addition, Kappa coefficient was included to evaluate the agreement between predicted and reference maps beyond chance, providing a robust measure of classification reliability. Together, these metrics capture not only pixel-wise accuracy but also classification consistency and robustness, which are essential for ensuring that the resulting land-cover maps can serve as reliable inputs for subsequent urban change and disturbance analysis.”

(6) *Q: How does the study handle edge cases where certain categories of land cover may have limited training data?*

A: We thank the reviewer for this question. We would like to clarify that due to our strict quality control during the data construction phase, our datasets are relatively balanced and do not suffer from severe edge cases or data scarcity issues.

Nevertheless, to pursue optimal segmentation performance—especially to ensure high robustness when handling complex geometric boundaries (such as thin roads)—we proactively adopted a composite loss function. The introduction of Dice Loss was not intended to compensate for data defects, but rather served as an optimization strategy to further enhance the model's ability to capture fine-grained details and prevent any potential pixel-level bias. This ensures that the model maintains superior performance even in rare, challenging scenarios.

The revisions can be found in Section 2.6(Lines 310-315) of the revised manuscript.

“A composite loss function combining categorical cross-entropy and Dice loss was adopted, with equal weighting, to balance classification accuracy, improve the recognition of hard samples, and optimize the segmentation of small target regions. This design also helps mitigate edge cases caused by class imbalance and limited training samples for certain land-cover categories, particularly for thin structures such as roads and fragmented greenbelts.”

(7) *Q: (Lines 285-315) what are the specific computational complexities of UASFNet compared to the other models presented in Table 5? How are computational costs justified relative to the performance gains?*

A: We thank the reviewer for the inquiry regarding computational complexity. The specific complexity metrics (such as parameters and FLOPs) for UASFNet compared to other models are detailed in Table 5.

Regarding the justification of these costs relative to performance gains, we have clarified in the text that the trade-off is favorable. While there is a moderate increase in computational cost, we believe it is fully justified by the consistent and non-trivial accuracy gains achieved across all datasets. High accuracy is critical for ensuring the reliability of large-area and multi-temporal urban change analysis, outweighing the incremental computational expense.

The revisions can be found in Section 4.1 (Lines 481-492) of the revised manuscript:

“From the perspective of model efficiency and performance balance, UASFNet demonstrates clear advantages over existing deep learning–based segmentation approaches. As summarized in Table 5, the proposed model achieves superior segmentation accuracy without relying on excessively large parameter sizes or computational costs. This balanced performance can be attributed to the Urban Adaptive Shared-feature Attention (UASF) module, which promotes effective semantic information sharing across multiple land cover categories while suppressing redundant feature propagation. As a result, UASFNet is able to extract more compact and discriminative representations, improving recognition performance under comparable computational conditions.All models were trained and evaluated on the same datasets and data partitions to ensure a controlled and fair comparison across different urban scenarios. The moderate increase in computational cost is justified by the consistent and non-trivial accuracy gains achieved across all datasets, which are critical for reliable large-area and multi-temporal urban change analysis.

Table 5 Model Efficiency and Performance Comparative Analysis

| Method | FLOPs (G) | Param.(M) | mIoU (%) |
| --- | --- | --- | --- |
| ResNet | 23.5 | 47.43 | 84.41 |
| UNetformer | 30.17 | 61.59 | 87.34 |
| CMTFNet | 76.41 | 96.14 | 90.30 |
| CM-Unet | 33.26 | 64.02 | 89.27 |
| MFNet | 54.39 | 67.72 | 87.45 |
| UASFNet (Ours) | 30.925 | 79.88 | 91.52 |

”

(8) *Q: In Table 2, what specific criteria were used to classify the models into "mainstream," and could this bias the results?*

A: Thank you very much for your valuable comment. We have carefully reviewed the terminology used in the manuscript and agree that the term "mainstream" might be interpreted as subjective or imprecise.

To avoid any ambiguity and eliminate potential bias, we have replaced this term with "representative" in the revised manuscript. These models were selected because they represent key milestones across different technical paradigms (e.g., CNN-based vs. Transformer-based approaches) within the field. This change clarifies our intention to provide a fair comparison against widely recognized benchmarks rather than a biased selection.

The revision can be found in Section 3.1 (Line 325-327) of the revised manuscript.

“For performance evaluation, UASFNet was compared with several representative deep learning–based semantic segmentation models commonly used in urban land-cover mapping, including ResNet(Liu and Goh, 2025), UNetformer(Wang et al., 2022), CMTFNet(Wu et al., 2023), CM-Unet(Liu et al., 2024), and MFNet(Ma et al., 2025).

To validate the overall performance of the proposed UASFNet model, comparative experiments were conducted on three representative urban remote sensing datasets—Langfang, Potsdam, and Guiyang—against five representative models.

(2) Across three representative datasets, UASFNet achieved mean mIoU values of 91.52%, 93.31%, and 88.90%, respectively—significantly outperforming existing representative models in the recognition accuracy of buildings, greenbelts, and roads—demonstrating strong cross-domain generalization capability.”

(9) *Q: (Lines 324-372) In Figure 5, how were the multi-temporal classification results verified? What controls or validation processes were in place during the mapping?*

A: We thank the reviewer for the question regarding the verification of multi-temporal classification results. To visually demonstrate the reliability of our results and provide an effective validation control, we have updated and expanded the relevant visualization in the revised manuscript (now Figure 9).

Figure 9 presents a "Large range of recognition effect comparison diagram in Langfang." By contrasting our classification results with actual ground features across a broad area, this figure serves as a direct visual verification of the model's accuracy and consistency in handling large-scale, multi-temporal urban mapping. This large-range comparison complements our quantitative metrics (such as mIoU) and acts as a crucial quality control step.

The revision can be found in Section 4.1 (Fig. 9) of the revised manuscript:.

“Fig.9 Large range of recognition effect comparison diagram in Langfang

”

(10) *Q: For Figure 6 and the corresponding discussion, what were the specific methodologies used to calculate the changes in impervious surfaces and green space? Could there have been biases in the data collection that affect these classifications?*

A: Thank you very much for your valuable comment. We have carefully revised the manuscript according to your suggestions.

We thank the reviewer for the attention to the details of the change analysis.

Regarding the specific methodology, we have clarified in the text that the changes in impervious surfaces and greenbelts were derived by aggregating class-wise pixel areas directly from the multi-temporal classification maps. This is a deterministic statistical approach, ensuring that the results accurately reflect the physical quantities represented in the classification outputs.

Regarding potential data collection biases, we controlled for this through a rigorous standardization process. As mentioned earlier, we applied unified acquisition protocols and preprocessing steps (such as geometric co-registration and normalization) across all datasets. Furthermore, since the calculation is based on full-coverage classification results rather than sampling, the impact of data collection bias on the final trend analysis is minimized.

The revisions can be found in Section 3.2 (Lines 378-383) of the revised manuscript:

“Fig. 6 illustrates the changes in the proportions of roads, buildings, greenbelts, and impervious surfaces across 34 subregions of the Langfang Economic Development Zone between 2017 and 2023. Impervious surface and greenbelt changes were derived by aggregating class-wise pixel areas from the multi-temporal classification maps. Overall, the study area experienced substantial land surface transformation, characterized by intensified imperviousness, a reduction in greenbelt, and a general expansion of built-up areas.

Fig. 6. Comparative charts of multiple indicators across the 34 subregions of the Langfang Economic ”

(11) *Q: Regarding Figure 7, can you elaborate on the significance of the spatial distribution patterns observed? How might these patterns inform future urban planning initiatives?*

A: We thank the reviewer for the insightful question regarding the interpretation of spatial distribution patterns and their implications for urban planning. We agree that elaborating on these patterns significantly enhances the practical value of our study.

In the revised manuscript, we have expanded the discussion on the spatially heterogeneous patterns observed in Figure 7. We highlighted that urban land cover change is not uniformly distributed but tends to concentrate in areas with intensive development activities. Furthermore, the co-occurrence of disturbances in buildings, roads, and greenbelts suggests that urban expansion exhibits a structured spatial organization rather than random dispersion.

Regarding the implications for future planning, we explained how these patterns serve as valuable spatial references. By identifying "development hotspots" and "relatively stable zones," these insights can inform more effective decision-making in terms of spatial prioritization and differentiated control strategies.

The revisions can be found in Section 3.3 (Lines 422-428) of the revised manuscript:

“These spatially heterogeneous distribution patterns observed in Fig. 7 indicate that urban land cover change is not spatially uniform but tends to concentrate in areas with intensive development activities. The co-occurrence and spatial coupling of building, road, and greenbelt disturbances suggest that urban expansion processes exhibit structured spatial organization rather than random dispersion. From an observational perspective, such spatial patterns provide useful spatial references for identifying development hotspots and relatively stable zones, which may inform future urban planning and land management decisions in terms of spatial prioritization and differentiated control.

Fig. 7 Spatial distribution of individual urban land cover disturbance indicators.”

(12) *Q: (Section 5, Lines 451-472) How can the findings of this study potentially influence policy on urban land use? What limitations in the data could potentially hinder this influence?*

A: We appreciate the reviewer’s inquiry regarding the potential influence of our findings on urban land use policy and the associated data limitations. This question is vital for clarifying the practical application value of our study.

In the revised manuscript, we have reorganized the relevant discussion (now in Section 4.2). First, regarding policy influence, we highlighted that the framework provides fine-grained classification and multi-scale change assessment. This "spatially explicit" information directly supports spatial governance, offering a scientific basis for evaluating land use intensity and monitoring ecological pressure during rapid urbanization, which is essential for formulating differentiated management policies.

Second, regarding data limitations, we maintained an objective perspective. We explicitly acknowledged that inconsistencies in image acquisition time and variations in spatial resolution across datasets could introduce uncertainty into long-term change interpretations. We transparently stated that these data-related constraints are factors that must be considered in practical applications and identified improving data consistency as a critical direction for future research.

The revisions can be found in Section 4.2 (Lines 524-541) of the revised manuscript:

“The proposed framework provides meaningful implications for urban land cover change analysis and spatial governance. By enabling fine-grained land cover classification and multi-scale change assessment, it facilitates a detailed characterization of urban expansion dynamics, infrastructure development, and greenbelt transformation. Such spatially explicit information supports a more refined evaluation of land use intensity and ecological pressure during rapid urbanization processes.

The integration of weighted disturbance indicators further enhances the interpretability of urban land cover change assessment. By synthesizing building, road, and greenbelt disturbances into a unified evaluation framework, the proposed approach enables consistent comparison across regions and time periods. This integrated perspective is particularly suitable for complex urban environments, where multiple land cover transitions often occur simultaneously and interactively rather than in isolation. Nevertheless, the practical application of the proposed framework may be influenced by limitations in the underlying data, such as inconsistencies in image acquisition time and variations in spatial resolution across different datasets, which could introduce uncertainty in long-term change interpretation. Addressing these data-related constraints through improved data consistency and preprocessing remains an important direction for future research.

Overall, the proposed framework demonstrates strong potential for long-term urban land cover monitoring and automated change assessment. Its data-driven and modular design allows flexible adaptation to different urban contexts and data sources, providing a scalable methodological foundation for future studies on urban dynamics and spatial governance.”

(13) *Q: What future applications are envisioned for the UASFNet framework, and how will evolving technology be incorporated into novel methodologies?*

A: We thank the reviewer for the forward-looking question regarding future applications and technological integration. We agree that discussing the scalability of the framework is essential for establishing its long-term value.

In the revised discussion, we have outlined the framework's promising applications in long-term urban land cover monitoring and automated change assessment, particularly in supporting fine-grained analysis of urban dynamics and spatial governance.

Regarding the integration of evolving technologies, we emphasized the framework's data-driven nature and modular design. This architectural choice ensures high flexibility, allowing the model to readily adapt to emerging technologies (such as improved backbones or novel data sources). Consequently, it provides a scalable and future-proof methodological foundation for continued research on urban dynamics.

The revisions and discussion can be found in Section 4.2 (Lines 524-541) of the revised manuscript.

“The proposed framework provides meaningful implications for urban land cover change analysis and spatial governance. By enabling fine-grained land cover classification and multi-scale change assessment, it facilitates a detailed characterization of urban expansion dynamics, infrastructure development, and greenbelt transformation. Such spatially explicit information supports a more refined evaluation of land use intensity and ecological pressure during rapid urbanization processes.

The integration of weighted disturbance indicators further enhances the interpretability of urban land cover change assessment. By synthesizing building, road, and greenbelt disturbances into a unified evaluation framework, the proposed approach enables consistent comparison across regions and time periods. This integrated perspective is particularly suitable for complex urban environments, where multiple land cover transitions often occur simultaneously and interactively rather than in isolation. Nevertheless, the practical application of the proposed framework may be influenced by limitations in the underlying data, such as inconsistencies in image acquisition time and variations in spatial resolution across different datasets, which could introduce uncertainty in long-term change interpretation. Addressing these data-related constraints through improved data consistency and preprocessing remains an important direction for future research.

Overall, the proposed framework demonstrates strong potential for long-term urban land cover monitoring and automated change assessment. Its data-driven and modular design allows flexible adaptation to different urban contexts and data sources, providing a scalable methodological foundation for future studies on urban dynamics and spatial governance.”

**Response to reviewer #3:**

(1) *Q: Title: The acronym UASFNet does not convey meaning to most readers and may obscure the paper’s intent. A more reader-focused alternative would be: “A Fine-grained Evaluation Framework for Urban Land Cover Change Based on Feature Monitoring with Remotely Sensed Imagery.” This revision communicates the achievement rather than emphasizing a technical acronym unfamiliar to the journal’s broad readership.*

A: We sincerely appreciate this constructive suggestion. We fully agree that avoiding obscure acronyms in the title is essential to clearly communicate the paper's intent and achievements to a broader readership.

We have fully accepted your advice and replaced the original title with the more descriptive and reader-focused alternative you proposed. The new title has been updated in the Title (Lines 1-2) of the revised manuscript:.

“A Fine-grained Evaluation Framework for Urban Land Cover Change Based on Feature Monitoring with Remotely Sensed Imagery”

(2) *Q: Abstract: The abstract outlines the general workflow but omits crucial information about the type of remote sensing imagery required. Given the diversity of sensors, platforms, pixel sizes, and spectral characteristics, this omission limits interpretability and reproducibility.The following components from the Case Study section should be incorporated into the abstract to clarify imagery requirements: Google Earth RGB imagery, 0.6 m resolution (Langfang dataset); Ultra–high-resolution TOP imagery, 5 cm GSD, multiple spectral combinations (Potsdam dataset); Standardized NAIP imagery for additional regional evaluation. Adding this information makes clear that the approach requires sub-meter to centimeter-scale RGB (and optionally IR) imagery characteristic of urban remote sensing segmentation tasks.*

A: We thank the reviewer for the suggestion regarding the completeness of the abstract. We fully agree that specifying the type, resolution, and spectral characteristics of the required remote sensing imagery is crucial for the interpretability and reproducibility of the study.

In the revised abstract, we have incorporated key descriptions regarding data characteristics, explicitly stating the use of "high-resolution sub-meter RGB urban remote sensing imagery." This revision synthesizes the common attributes of the datasets used in our case studies (e.g., Langfang and Potsdam) and clearly defines the data prerequisites (sub-meter to centimeter-scale) for the proposed approach.

The revision can be found in the Abstract (Lines 15-33) of the revised manuscript:

“Against the backdrop of accelerating global climate change and urbanization, urban land cover change has emerged as a critical indicator for understanding the dynamic evolution of cities and the transformation of urban ecosystems. This study proposes a data-driven framework for fine-scale urban land cover change assessment based on the UASFNet model, enabling high-precision evaluation of urban land cover dynamics. The approach first performs preprocessing and co-registration of bi-temporal remote sensing images from the study area, and applies the trained UASFNet model to identify urban land cover types and extract land cover information for each temporal phase. The Analytic Hierarchy Process (AHP) is then employed to determine the weights of various indicator factors. By integrating building disturbance, greenbelt disturbance, and road disturbance indices, the framework quantitatively evaluates the intensity of land cover change at both pixel and regional scales. Experimental results across three benchmark datasets, consisting of high-resolution sub-meter RGB urban remote sensing imagery, demonstrate that UASFNet achieves superior segmentation accuracy, with mean Intersection over Union (mIoU) values of 91.52%, 93.31%, and 88.90%, substantially outperforming several state-of-the-art baseline models. Spatial analysis of the Langfang urban area (2017–2023) reveals a marked increase in impervious surface coverage (+16.86%) and a sharp decline in greenbelt (−40%), with the urban landscape exhibiting a multi-core, belt-like expansion pattern oriented toward newly developed districts. The proposed framework not only enhances the interpretability and generalization of remote sensing models in complex urban environments but also provides a scalable analytical tool to support urban spatial planning, ecological conservation, and sustainable city governance.”

(3) *Q: Introduction: The final paragraph presents the study’s intended academic contributions, but these are not framed as testable scientific questions. For example, the introduction could explicitly pose questions such as:Does UASFNet significantly outperform established semantic segmentation methods (e.g., ResNet, UNetformer, MFNet) in boundary preservation and inter-class separability for urban land cover types?Does integrating AHP-derived indicator weights meaningfully improve the spatial coherence or interpretability of disturbance-level assessments?Can combined pixel-level and region-level analyses provide a measurably more accurate or actionable representation of urban land cover change than existing pixel-only approaches?Explicit questions would help readers understand what hypotheses the study is designed to test and how success is measured.*

A: We appreciate the reviewer's suggestion to explicitly frame the study around testable scientific inquiries. We agree that clearly defining the hypotheses is essential for helping readers understand the study's design and success metrics.

In response, we have refined the final paragraph of the Introduction. We have rephrased the contributions to explicitly align with the specific scientific questions raised by the reviewer. Specifically, the revised text clarifies that the study is designed to:

1. Systematically evaluate the effectiveness of UASFNet in enhancing boundary delineation and inter-class separability against baseline methods;

2. Demonstrate how the AHP-based integration scheme enables interpretable and spatially coherent change assessment;

3. Validate the advantages of the multi-scale framework (combining pixel-level and region-level analyses) in characterizing urban change patterns.

This revision ensures that the study's underlying hypotheses and validation goals are clearly presented. The revisions can be found in Section 1 (Lines 90-99) of the revised manuscript:.

“The potential academic contributions of this study are as follows: (i) An Urban Adaptive Shared-feature Attention Network (UASFNet) is developed for fine-grained urban land cover mapping, and its effectiveness in enhancing boundary delineation and inter-class separability is systematically evaluated against representative semantic segmentation baselines in complex urban scenes;(ii) A weighted disturbance integration scheme based on the Analytic Hierarchy Process (AHP) is introduced to fuse building, road, and greenbelt change indicators, enabling interpretable and spatially coherent urban land cover change assessment;(iii) A multi-scale evaluation framework combining pixel-level classification and region-level aggregation is proposed to improve the characterization and interpretability of urban land cover change patterns beyond pixel-only analyses. ”

(4) *Q: Methods: sub-heading UASFNet Mathematical Formulation- The core equations for the UASF module—semantic–structural consistency kernel, gradient-domain formulation, and global gating operator—are insufficiently explained for a general audience. The manuscript introduces functions (ϕ(X), φ(∇X), Θ(X), A(X)) without an illustrative example. The authors should include: A worked example: Starting from a raw image tile, show how features, gradients, embeddings, and fused representations are extracted and transformed through each stage.A schematic diagram showing how semantic and structural pathways merge and how gating weights are computed.Code availability: Since PLOS ONE emphasizes reproducibility, the authors should provide code or pseudocode to clarify the transformations embodied in Equations (2) and (3). Without such explanation, the method is not reproducible or interpretable by most readers.*

A: We thank the reviewer for the critical feedback regarding the interpretability and reproducibility of the methodology. We fully agree that a clear physical interpretation, accompanied by visual aids and explicit algorithmic steps, is essential for readers.

In response to your specific requests, we have made the following comprehensive revisions:

1.Detailed Explanation (Worked Example): We have added a comprehensive explanation in Section 2.3 (Lines 196-202) clarifying the physical rationale behind the semantic–structural consistency kernel, gradient-domain formulation, and global gating operator.

2.Pseudocode for Reproducibility: To fully address the reproducibility requirement of PLOS ONE, we have explicitly included Algorithm 1 in the revised manuscript. This pseudocode details the step-by-step transformations—from raw input features to the final fused representation—ensuring that the method is reproducible and interpretable.

The revisions and the added Algorithm 1 are as follows:.

“The semantic–structural consistency kernel encourages the alignment of semantic representations with geometric cues, which is beneficial for preserving object boundaries and reducing confusion between visually similar land cover classes. The gradient-domain formulation highlights local structural variations, such as edges and shape transitions, that are particularly relevant for fine-scale urban patterns. The global gating operator adaptively modulates the relative contributions of semantic and structural information across spatial locations, thereby promoting spatial coherence while limiting redundant feature propagation.

**Algorithm 1: Urban Adaptive Shared-feature Attention (UASF) Module.**

Input: Feature map X ∈ R^B×C×H×W^

Parameters: DWConv, PWConv, Sobel(·), FC1, FC2, Proj(·), σ(·), δ(·)

Output: Fused feature representation Y∈ R^B×C×H×W^

1: % Semantic Embedding Branch (Feature-domain)

2: E ← PWConv(DWConv(X))

3:

4: % Structural Modulation Branch (Gradient-domain)

5: Gx, Gy ← Sobel(X)

6: S ← sqrt(Gx ⊙ Gx + Gy ⊙ Gy + ε)

7: S ← Normalize(S)

8: 9: % Semantic-Structural Consistency Kernel

10: Z ← E ⊙ (1 + S) (⊙: element-wise multiplication)

11: Z ← Proj(Z)

12:

13: % Global Adaptive Gating

14: v ← GAP(X)

15: a ← δ(FC1(v))

16: g ← σ(FC2(a))

17: g ← Reshape(g) to R^B×C×1×1^

18:

19: % Final Fusion Output

20: Y ← g ⊙ Z + (1 − g) ⊙ X

21: return Y

”

(5) *Q: Methods: sub-heading Evaluation Framework - The manuscript states that “multiple metrics” are used—mIoU, accuracy, F1, Kappa, etc.—but does not clearly tie these metrics to specific stated objectives (boundary preservation, inter-class separability, semantic consistency). The connection between objectives and metrics must be explicit. Similarly, the land cover change evaluation framework (using AHP to weight building, road, and greenbelt disturbance indices) would benefit from: A clear workflow diagram; A rationale for indicator selection; An explanation of how errors in land cover classification propagate into disturbance-level scoring.*

A: We thank the reviewer for the detailed suggestions regarding the evaluation framework. We fully agree that explicitly linking metrics to objectives and visualizing the workflow are essential for methodological transparency.

In response to your comments, we have made the following revisions:

1. Linking Metrics to Objectives: We have clarified the specific role of each metric in Section 2.4. Specifically, mIoU is used to quantify overall pixel-level accuracy; Precision, Recall, and F1-score are employed to evaluate class-wise discrimination (particularly for structurally complex categories); and the Kappa coefficient is included to measure classification reliability beyond random chance.

2.Visualization and Rationale of the AHP Framework:

(1)Workflow Diagram: We refer the reviewer to the updated Figure 1 (Steps 4–6) in the revised manuscript. This figure now provides a clear schematic of the entire workflow, from "Index Selection" to "Weight Determination (AHP)" and "Urban Disturbance Zoning".

(2)Rationale: We clarified that building, road, and greenbelt disturbances were selected as indicators because they represent the physical intensity, connectivity, and ecological impact of urban expansion, respectively.

(3)Error Propagation: We acknowledged that classification errors can propagate into disturbance scoring. However, by adopting a weighted multi-indicator fusion scheme, the impact of local errors from single categories is mitigated, ensuring the robustness of the comprehensive assessment.

The textual revisions regarding the metrics can be found in Section 2.4 (Lines 217-224) of the revised manuscript:.

“The mean Intersection over Union (mIoU) was used as the primary metric to quantify overall pixel-level classification accuracy across land-cover classes. Precision, recall, and F1-score were employed to further characterize class-wise discrimination performance, particularly for imbalanced or structurally complex categories. In addition, Kappa coefficient was included to evaluate the agreement between predicted and reference maps beyond chance, providing a robust measure of classification reliability. Together, these metrics capture not only pixel-wise accuracy but also classification consistency and robustness, which are essential for ensuring that the resulting land-cover maps can serve as reliable inputs for subsequent urban change and disturbance analysis.

Fig. 1 Overall framework of the proposed fine-scale urban land cover change assessment method.

This study proposes a data-driven framework for fine-scale urban land cover change assessment based on the UASFNet architecture (Fig. 1). The framework consists of four main stages. First, bi-temporal high-resolution remote sensing images of the study area are preprocessed and co-registered to ensure spatial consistency between different observation periods. These images serve as the input data for pixel-level land cover classification. Second, the trained UASFNet model is applied to the preprocessed imagery to identify urban land cover types and generate fine-grained land cover maps for each temporal phase. The resulting classification outputs provide the basis for subsequent change analysis. Third, building disturbance, greenbelt disturbance, and road disturbance indicators are derived from the classification results. The Analytic Hierarchy Process (AHP) is employed to determine the relative weights of these indicators, enabling the integration of heterogeneous change information. Finally, the weighted disturbance indicators are aggregated to evaluate the comprehensive intensity and spatial distribution of urban land cover change across the study area.”

(6) *Q: Experimental Detail: This subsection belongs more naturally in Methods under a heading such as:“Computational Environment and Data Augmentation Strategy.”The description of the GPU, Python environment, and augmentation techniques is overly brief and should be expanded with parameter values and more details for helping others evaluate, test, replicate, improve your work. There are standard components of deep learning methodology and are necessary for reproducibility.*

A: We appreciate the reviewer’s suggestion regarding the manuscript structure and experimental details. We agree that placing this subsection within the Methodology section and providing precise hardware specifications and parameter settings is essential for reproducibility.

Following your advice, we have moved these details to Section 2.6 of the revised manuscript. We have significantly expanded the description to include the specific computational environment (e.g., Windows 11, NVIDIA RTX 4090 GPU, PyTorch framework). Furthermore, we have specified exact training hyperparameters, including a batch size of 4, the use of the Adam optimizer with an initial learning rate of 1e-4, and specific data augmentation parameters (e.g., rotation within ±15°). We also detailed the configuration of the composite loss function. These additions provide all necessary information for other researchers to evaluate and replicate our work.

The revisions can be found in Section 2.6 (Lines 300-315) of the revised manuscript:

“In this study, the bi-temporal remote sensing interpretation module was implemented using Python and the PyTorch framework. All model training and evaluation were conducted on a Windows 11 operating system equipped with an NVIDIA® GeForce RTX™ 4090 GPU, ensuring efficient computational resource support. For data augmentation, random horizontal and vertical flipping, as well as rotation within ±15°, were applied to increase the diversity of the training dataset, thereby enhancing the model’s ability to generalize to unseen data.

During the training phase, the batch size is set to 4 based on the GPU memory capacity and model complexity, with an initial learning rate of 1e-4. The Adam optimizer is used to optimize the training process by automatically adjusting the gradient mean and variance for each parameter. The total number of training epochs is set to 300 to ensure that the model converges after sufficient learning. In classification, the model is divided into four categories (building, road, greenbelt, and others). A composite loss function combining categorical cross-entropy and Dice loss was adopted, with equal weighting, to balance classification accuracy, improve the recognition of hard samples, and optimize the segmentation of small target regions. This design also helps mitigate edge cases caused by class imbalance and limited training samples for certain land-cover categories, particularly for thin structures such as roads and fragmented greenbelts.”

(7) *Q: Model Performance: This section actually constitutes Results, not a case study. The models used for comparison—ResNet, UNetformer, CMTFNet, CM-Unet, MFNet—should be introduced earlier in the Methods section under: “Benchmark Models and Comparative Evaluation Design.” Currently, the paper reads as though the evaluation details appear only after results, which makes the structure confusing.*

A: We appreciate the reviewer's suggestion regarding the manuscript structure. We fully agree that introducing the benchmark models within the Methods section prior to presenting the results significantly improves the logical flow of the paper.

Following your advice, we have incorporated the description of "Benchmark Models and Comparative Evaluation Design" into Section 2.6 of the revised manuscript. Here, we formally introduced the representative models used for comparison (including ResNet, UNetformer, CMTFNet, CM-Unet, and MFNet), ensuring that the evaluation setup is clearly established before the Results section.

(8) *Q: Case Study Results: Land cover change interpretation: The manuscript presents tables of pixel-level and region-level disturbance statistics and interprets the spatial implications, but this should be more clearly labeled as Results, not embedded inside the case study narrative. The interpretation itself is appropriate and meaningful, showing both fine-scale and aggregated change patterns.*

A: We appreciate the reviewer's suggestion regarding the standardization of the section structure. We fully agree that explicitly labeling this section as a standard "Results" section, rather than embedding it within a case study narrative, significantly enhances the clarity and academic rigor of the manuscript.

Following your advice, we have renamed the main heading of Section 3 to "Results" and reorganized the subsections to clearly distinguish between model performance, classification outputs, and spatial change analysis.

The revised structure is as follows (see Section 3.1 - 3.4 of the revised manuscript):

“3 Results , 3.1. Model performance , 3.2. Multi-Temporal Urban Land Cover Classification Results, 3.3. Spatial Distribution of Urban Land Cover Change Evaluation Indicators, 3.4. Analysis of Urban Land Cover Change Evaluation Results”

(9) *Q: Weaknesses: The evaluation is not explicitly tied to the stated objectives (e.g., boundary preservation, structural consistency). No statistical tests (e.g., paired t-tests on pixel accuracy, variance analyses across tiles) are reported to support claims of significance. The study does not examine failure cases, computational complexity trade-offs, or robustness to noise, which would strengthen claims of generalizability. The evaluation does not address whether the AHP-based disturbance metrics produce more accurate or actionable land cover change assessments than alternative weighting methods.*

A: Thank you very much for your valuable comment. We have carefully revised the manuscript according to your suggestions.

The revisions can be found in Section 3.1(Lines 321-324), 3.2(Lines 362-365), 3.3(Lines 403-405), 3.4(Lines 432-435) of the revised manuscript.

“This section evaluates the classification performance of the proposed UASFNet in comparison with five models introduced in the Methods section across different datasets. By reporting both category-level and overall performance, this analysis aims to provide an objective assessment of the relative effectiveness of UASFNet for fine-grained urban land cover classification.

This subsection presents the multi-temporal urban land cover classification results derived from UASFNet for different observation periods. By analyzing classification maps from multiple years, this section examines the temporal consistency of the model outputs and highlights major patterns of land cover change associated with urban development.

Based on the multi-temporal classification results, this subsection analyzes the spatial distribution of urban land cover change evaluation indicators. The analysis focuses on the spatial patterns and heterogeneity of building, road, and greenbelt changes at the pixel and local scales.

This subsection provides a comprehensive analysis of urban land cover change evaluation results by integrating multiple indicators into an overall disturbance assessment. The analysis is conducted at both pixel and regional levels to examine how localized changes aggregate into broader urban transformation patterns.

”

(10) *Q: Conclusion on Objectivity: The manuscript does provide a comparative performance assessment, but it is only partially objective and not fully aligned with the evaluation goals described in the Methods section. Strengthening the study would require tighter linkage between model objectives and metrics, inclusion of statistical validation, and clearer articulation of how the proposed framework improves land cover change interpretation beyond classification accuracy alone.*

A: We thank the reviewer for summarizing the weaknesses of the study. We have taken these points very seriously and have systematically revised and restructured the manuscript to address them comprehensively.

Specifically, we have reorganized the Results (Section 3) and Discussion (Section 4) sections to ensure that the evaluation is explicitly tied to our stated objectives and covers the missing analytical dimensions:

Section 3.1 focuses on an objective assessment of model performance, utilizing the multi-dimensional metrics clarified in the Methodology;

Section 3.2 examines the temporal consistency of multi-temporal classification outputs;

Section 3.3 and 3.4 analyze the spatial distribution patterns and the effectiveness of the integrated disturbance indicators;

Section 4.2 further discusses the implications of the comprehensive evaluation results.

The introductory paragraphs of the revised sections are provided below to demonstrate the strengthened evaluation logic:.

“This section evaluates the classification performance of the proposed UASFNet in comparison with five models introduced in the Methods section across different datasets. By reporting both category-level and overall performance, this analysis aims to provide an objective assessment of the relative effectiveness of UASFNet for fine-grained urban land cover classification.

This subsection presents the multi-temporal urban land cover classification results derived from UASFNet for different observation periods. By analyzing classification maps from multiple years, this section examines the temporal consistency of the model outputs and highlights major patterns of land cover change associated with urban development.

Based on the multi-temporal classification results, this subsection analyzes the spatial distribution of urban land cover change evaluation indicators. The analysis focuses on the spatial patterns and heterogeneity of building, road, and greenbelt changes at the pixel and local scales.

This subsection provides a comprehensive analysis of urban land cover change evaluation results by integrating multiple indicators into an overall disturbance assessment. The analysis is conducted at both pixel and regional levels to examine how localized changes aggregate into broader urban transformation patterns.”

**Response to reviewer #4:**

(1) *Q: Introduction/Literature Review: While a general categorization of research in the field is provided, a more focused review of studies directly related to the presented methodology is needed. This would establish a clearer baseline and context, allowing readers to better assess the novelty and contribution of the proposed method relative to existing work.*

A: We appreciate the reviewer's suggestion regarding the focus of the literature review. We fully agree that a more specific review of studies directly related to our methodology is essential for establishing a clear baseline and highlighting the novelty of our work.

In the revised manuscript, we have strengthened the literature review by explicitly addressing the disconnect between current fine-grained land cover mapping and change assessment efforts. We highlighted that existing deep learning models are often evaluated solely on pixel-level accuracy, while indicator-based change assessment frameworks typically rely on coarse-resolution inputs. This discussion provides a clear context and rationale for our proposed framework, which bridges the gap by integrating boundary-aware semantic segmentation with weighted, interpretable disturbance assessment.

The revisions can be found in Section 1 (Lines 70-87) of the revised manuscript:“Urban land cover change not only reflects the rate and direction of spatial expansion but is also directly linked to the evolution of ecological security patterns and the vulnerability of urban systems(Luo et al., 2023; Yang et al., 2023; Zhong et al., 2024). In existing studies, mainstream assessment approaches typically rely on indicator-based frameworks and weighting analyses(Liu et al., 2021)—employing techniques such as the AHP(Karimpour et al., 2014), Entropy Weight Method(Baemez, 2025), and Fuzzy Comprehensive Evaluation(Wang et al., 2025)—to quantify the magnitude of land-use change and its ecological implications. These methods have been widely applied in regional-scale analyses of urban vulnerability, sustainability, and landscape pattern dynamics(Wang et al., 2024). However, conventional approaches that depend on statistical data or low spatiotemporal resolution imagery are insufficient to capture subtle surface disturbances and spatial heterogeneity associated with urbanization processes. By leveraging pixel-level land cover change results derived from deep learning models as the primary analytical driver, a fine-scale urban surface change assessment framework can be established to accurately identify and quantify urban land cover dynamics. However, existing studies often treat fine-grained land cover mapping and change assessment as relatively independent tasks. Deep learning–based segmentation models are mainly evaluated in terms of pixel-level accuracy, while indicator-based change assessment frameworks commonly rely on coarse-resolution or aggregated inputs. As a result, the linkage between boundary-aware urban land cover recognition and disturbance-level interpretation remains weak, particularly in dense urban cores. This gap highlights the need for an integrated framework that explicitly connects fine-scale semantic segmentation with weighted, interpretable urban land cover change assessment.”

(2) *Q: Research Objectives: The objectives stated at the end of the introduction should more explicitly highlight and emphasize the novel aspects of the proposed approach compared to previous studies.*

A: We appreciate the reviewer's suggestion to explicitly highlight the novel aspects of our research objectives. We fully agree that clearly articulating the unique contributions of our approach relative to existing studies is essential for establishing the academic value of the paper.

In response, we have refined the final paragraph of the Introduction to strictly define our research objectives, with a specific emphasis on three core novel aspects:

1.Model Novelty: The development of UASFNet, designed to address the challenges of boundary delineation and inter-class separability in complex urban scenes (improving upon representative baselines);

2.Methodological Novelty: The introduction of an AHP-based weighted disturbance integration scheme, enabling interpretable and spatially coherent change assessment;

3.Framework Novelty: The proposal of a multi-scale evaluation framework that combines pixel-level and region-level analyses to better characterize urban change patterns (going beyond pixel-only analyses).

The revisions can be found in Section 1 (Lines 88-103) of the revised manuscript:

“This study aims to propose a data-driven framework for fine-scale urban land cover change assessment based on UASFNet, designed to support urban spatial governance and ecological optimization decisions while revealing the dynamic evolution of surface systems during urbanization. The potential academic contributions of this study are as follows: (i) An Urban Adaptive Shared-feature Attention Network (UASFNet) is developed for fine-grained urban land cover mapping, and its effectiveness in enhancing boundary delineation and inter-class separability is systematically evaluated against representative semantic segmentation baselines in complex urban scenes;(ii) A weighted disturbance integration scheme based on the Analytic Hierarchy Process (AHP) is introduced to fuse building, road, and greenbelt change indicators, enabling interpretable and spatially coherent urban land cover change assessment;(iii) A multi-scale evaluation framework combining pixel-level classification and region-level aggregation is proposed to improve the characterization and interpretability of urban land cover change patterns beyond pixel-only analyses. The structure of this paper is organized as follows: Section 2 describes the materials and methods, including the proposed methodological framework and data preparation. Section 3 presents the results of urban land cover classification and change analysis based on the UASFNet model. Section 4 discusses the performance, applicability, and implications of the proposed approach. Section 5 concludes the study.”

(3) *Q: Lines 111-113 / Overall Procedure: The description of the proposed method is somewhat vague. The specific steps of the pipeline should be clearly delineated. For each stage, it should be explicitly stated what processing is applied to the input, what the output is, and why this output is fed into the next stage. This lack of clarity is also reflected in the provided flowchart, making it difficult for the reader to follow the logical flow of the method.*

A: We appreciate the reviewer's feedback regarding the clarity of the overall procedure. We fully agree that explicitly delineating the input, processing logic, and output for each stage of the pipeline is essential for understanding the proposed method.

In response, we have rewritten the "Overall Procedure" subsection to align strictly with the updated flowchart (Figure 1). We adopted a step-by-step description in the revised manuscript to clearly define the workflow:

1.Preprocessing: Specifying bi-temporal images as input and co-registration as the process to ensure spatial consistency;

2.Classification: Applying UASFNet to generate fine-grained land cover maps, which serve as the basis for change analysis;

3.Indicator Calculation: Deriving disturbance indicators from classification results and employing AHP for weighting;

4.Comprehensive Evaluation: Aggregating weighted indicators to evaluate overall change intensity.

The revisions can be found in Section 2.1 (Lines 109-120) of the revised manuscript:.

“This study proposes a data-driven framework for fine-scale urban land cover change assessment based on the UASFNet architecture (Fig. 1). The framework consists of four main stages. First, bi-temporal high-resolution remote sensing images of the study area are preprocessed and co-registered to ensure spatial consistency between different observation periods. These images serve as the input data for pixel-level land cover classification. Second, the trained UASFNet model is applied to the preprocessed imagery to identify urban land cover types and generate fine-grained land cover maps for each temporal phase. The resulting classification outputs provide the basis for subsequent change analysis. Third, building disturbance, greenbelt disturbance, and road disturbance indicators are derived from the classification results. The Analytic Hierarchy Process (AHP) is employed to determine the relative weights of these indicators, enabling the integration of heterogeneous change information. Finally, the weighted disturbance indicators are aggregated to evaluate the comprehensive intensity and spatial distribution of urban land cover change across the study area.”

(4) *Q: Justification of Weighting: The rationale and importance of using a weighting scheme (Lines 111-113, Line 180) are not sufficiently explained. A clear justification is required: Why is weighting crucial for identifying the target changes in this specific context? What problem does it solve that non-weighted approaches do not?*

A: We thank the reviewer for the inquiry regarding the rationale of the weighting scheme. We fully agree that justifying "why weighting is necessary" is crucial for the scientific validity of the method.

To address this, we have clarified the necessity of the weighting mechanism in the text. We pointed out that direct aggregation implicitly assumes "equal importance" across all land-cover components. This assumption is often flawed in complex urban environments, as it may mask dominant drivers of urban change, thereby reducing interpretability.

In contrast, by introducing weighting, we can explicitly reflect the differentiated contributions of building, road, and greenbelt disturbances to the overall assessment. This revision clearly articulates the specific problem that our weighting scheme aims to solve.

The revisions can be found in Section 2.5 (Lines 242-244, 268-271) of the revised manuscript:

“Direct aggregation of these disturbance indicators would implicitly assume equal importance across different land-cover components, which may mask dominant drivers of urban land cover change and reduce interpretability in dense urban environments.

In this study, weighting is introduced to explicitly reflect the differentiated contributions of building, road, and greenbelt disturbances to the overall urban land cover change assessment.”

(5) *Q: Methodology Subsections & Citations: The subsections under the methodology present various formulas and relationships without any citations to prior work. It appears that all elements were developed by the authors, which is unusual for a standard data mining/remote sensing workflow. Relevant sources for established techniques should be cited.*

A: We appreciate the reviewer's important reminder regarding citation standards. We fully agree that established techniques and formulas within the methodology should be properly cited to distinguish standard methods from the novel contributions of this study.

Following your suggestion, we have supplemented the revised manuscript with relevant citations:

When describing the preprocessing pipeline (e.g., geometric co-registration, resampling, and normalization), we added citations (Li et al., 2023; Wang et al., 2025) to acknowledge these as standard procedures.

When presenting the calculation methods for evaluation metrics (Table 1), we added the citation (Wu et al., 2023) to provide a reference for the metrics used.

(6) *Q: Subsection Structure (Lines 234-236): The inclusion of several very brief subsections with minimal content is not effective. Each subsection should contain substantive material related to its title.*

A: We appreciate the reviewer's feedback regarding the subsection structure. We fully agree that having very brief subsections with minimal content limits the effectiveness and depth of the presentation. Each subsection should indeed contain substantive material aligned with its title.

To address this, we have substantively expanded and refined the subsections within Section 3 (Results). Specifically, we have added comprehensive introductory paragraphs to Sections 3.1, 3.2, 3.3, and 3.4. These additions clearly define the evaluation objectives, analytical scope, and logical framework for each subsection, ensuring that each part is supported by substantive analysis rather than brief descriptions.

The revised introductory content for these subsections is as follows:

“This section evaluates the classification performance of the proposed UASFNet in comparison with five models introduced in the Methods section across different datasets. By reporting both category-level and overall performance, this analysis aims to provide an objective assessment of the relative effectiveness of UASFNet for fine-grained urban land cover classification.

This subsection presents the multi-temporal urban land cover classification results derived from UASFNet for different observation periods. By analyzing classification maps from multiple years, this section examines the temporal consistency of the model outputs and highlights major patterns of land cover change associated with urban development.

Based on the multi-temporal classification results, this subsection analyzes the spatial distribution of urban land cover change evaluation indicators. The analysis focuses on the spatial patterns and heterogeneity of building, road, and greenbelt changes at the pixel and local scales.

This subsection provides a comprehensive analysis of urban land cover change evaluation results by integrating multiple indicators into an overall disturbance assessment. The analysis is conducted at both pixel and regional levels to examine how localized changes aggregate into broader urban transformation patterns.”

(7) *Q: Specific Comments on Figures and Tables: Figure 1: The caption should be more detailed and specific, describing what the figure illustrates. This applies to all figure and table captions throughout the manuscript. Figure 3: The defined legend for this figure appears to be incorrect. Please verify and correct it. Table 2: It is unclear why all values for the proposed method are highlighted with a different color. Typically, only the best-performing values (e.g., highest accuracy) should be bolded or highlighted for clear comparison. Table 3: The methodology for generating the rankings is not described. How were these rankings obtained? Was a single expert used, or multiple experts? If multiple, how was consensus reached? This information is essential for assessing the validity of the comparison.*

A: We thank the reviewer for the detailed scrutiny of the figures and tables. We fully agree that precise captions, standardized formatting, and clear methodological explanations are essential for the quality of the manuscript.

We have addressed your specific comments point-by-point:

1.Captions: We have thoroughly reviewed and revised the captions for all figures and tables throughout the manuscript to be more detailed and specific. The updated captions are listed below (see Section 2 & 3 of the revised manuscript).

2.Figure 3 Legend: We have verified and corrected the incorrect legend in Figure 3.

3.Table 3 Methodology: We have clarified that the rankings in Table 3 are derived from an Expert-based AHP judgment matrix. We added details in the text explaining that this matrix was constructed based on consultation and consensus among domain experts.

The revised captions are as follows:

“

Fig. 1 Overall framework of the proposed fine-scale urban land cover change assessment method.

Fig. 3 Example image patches and corresponding ground truth labels from the Potsdam dataset.

Fig. 4 Visual comparison of urban land cover classification results produced by different models on the the Potsdam dataset

Table 2 Quantitative performance comparison of different semantic segmentation models on three urban remote sensing datasets.

Fig. 5 Multi-temporal urban land cover classification results for the Langfang Economic Development Zone.

Fig. 7 Spatial distribution of individual urban land cover disturbance indicators.

Fig. 8 Spatial distribution of comprehensive urban land cover change intensity.

Table 3 Expert-based AHP judgment matrix for urban land-cover disturbance indicators”
